# Impaired glymphatic system revealed by DTI-ALPS in cerebral palsy due to periventricular leukomalacia: relation with brain lesion burden and hand dysfunction

**DOI:** 10.1007/s00234-023-03269-9

**Published:** 2023-12-22

**Authors:** Yu Yin, Ying Peng, Lisha Nie, Xianjun Li, Yaqiong Xiao, Haoxiang Jiang, Lei Gao, Heng Liu

**Affiliations:** 1 Department of Radiology, Affiliated Hospital of Zunyi Medical University, Medical Imaging Center of Guizhou Province, Zunyi, China; 2 Department of Radiology, Second Xiangya Hospital of Central South University, Changsha, 410011 Hunan Province China; 3 GE Healthcare, MR Research China, Beijing, China; 4 Department of Radiology, The First Affiliated Hospital of Xi’an Jiaotong University, Xi’an, China; 5 Center for Language and Brain, Shenzhen Institute of Neuroscience, Shenzhen, China; 6 Department of Radiology, Wuxi Children’s Hospital Affiliated to Nanjing Medical University, Wuxi, China; 7 Department of Radiology, Zhongnan Hospital of Wuhan University, Wuhan City, Hubei Province China

**Keywords:** Cerebral palsy, Periventricular leukomalacia, Glymphatic system, Brain lesion burden, Hand dysfunction

## Abstract

**Purpose:**

Preterm children with cerebral palsy (CP) often have varying hand dysfunction, while the specific brain injury with periventricular leukomalacia (PVL) cannot quite explain its mechanism. We aimed to investigate glymphatic activity using diffusion tensor image analysis along the perivascular space (DTI-ALPS) method and evaluate its association with brain lesion burden and hand dysfunction in children with CP secondary to PVL.

**Methods:**

We retrospectively enrolled 18 children with bilateral spastic CP due to PVL and 29 age- and sex-matched typically developing controls. The Manual Ability Classification System (MACS) was used to assess severity of hand dysfunction in CP. A mediation model was performed to explore the relationship among the DTI-ALPS index, brain lesion burden, and the MACS level in children with CP.

**Results:**

There were significant differences in the DTI-ALPS index between children with CP and their typically developing peers. The DTI-ALPS index of the children with CP was lower than that of the controls (1.448 vs. 1.625, *P* = 0.003). The mediation analysis showed that the DTI-ALPS index fully mediated the relationship between brain lesion burden and the MACS level (*c*′ = 0.061, *P* = 0.665), explaining 80% of the effect.

**Conclusion:**

This study provides new insights into the neural basis of hand dysfunction in children with CP, demonstrating an important role of glymphatic impairment in such patients. These results suggest that PVL might affect hand function in children with CP by disrupting glymphatic drainage.

## Introduction

Periventricular leukomalacia (PVL) is a common type of brain injury in premature infants and has been widely recognized as a major cause of spastic cerebral palsy (CP) [1, 2]. PVL is believed to result from developmental immaturity, ischemia-hypoxia, and perinatal infection, leading to cell death, excitotoxicity, free radical accumulation, energy failure, and neuroinflammation [3–5]. These processes contribute to focal necrosis in the periventricular white matter and diffuse reactive gliosis. Advancements in structural magnetic resonance imaging (MRI) have enhanced our understanding of CP secondary to PVL. In addition to periventricular white matter, structural damages in subcortical white matter, cortical gray matter, and deep gray matter were also frequently observed in CP [6–8]. These primary injuries disturb the development of preterm infants, leading to impaired neuronal migration, differentiation, or maturation, resulting in severe neurological deficits mainly in motor dysfunction [9]. The extent and location of brain damage greatly vary among children with CP, leading to diverse clinical symptoms, such as hand dysfunction [10, 11]. Although previous semi-quantitative magnetic resonance studies have established a correlation between lesion burden and functional abilities in children with CP [12–14], it is widely acknowledged that brain lesion burden alone does not fully explain or predict motor outcomes, suggesting the involvement of other underlying mechanisms.

The glymphatic system, a recently discovered “waste clearance” system in the brain, plays a crucial role in clearing metabolites and toxic wastes from the brain [15]. Cerebrospinal fluid flows into the brain parenchyma through the arterial perivascular space, undergoes rapid exchange with interstitial fluid via aquaporin-4 (AQP4) in astrocytes, and eventually drains into the venous perivascular space, meninges, and cervical lymphatics. AQP4 polarization ensures the normal functioning of the glymphatic system [16]. Several studies on AQP4-null mice have demonstrated that the loss of perivascular AQP4 impairs glymphatic transport and clearance capacity [15, 17, 18]. Reactive astrocyte gliosis, a common feature in various central nervous system disorders, reduces AQP4 polarization and consequently impairs the glymphatic system [19–21]. While the mechanism of PVL remains incompletely understood, reactive astrogliosis plays a significant role in its development. Given that astrocytes are integral to the glymphatic system, a potential link between glymphatic dysfunction and PVL can be hypothesized.

The existence of the glymphatic pathway was initially described in rodent brains, and subsequent intrathecal contrast-enhanced MRI studies confirmed its presence in the human brain [15, 22, 23]. However, the invasive nature and limited clinical application of intrathecal contrast administration necessitate the establishment of noninvasive methods to assess human glymphatic function. Recently, Taoka et al. proposed the use of diffusion tensor imaging along the perivascular space (DTI-ALPS) as a noninvasive means to estimate the human glymphatic activity [24]. DTI-ALPS assesses the movement of water molecules in different directions along the perivascular space by measuring diffusivity, a critical process within the glymphatic pathway. Studies have shown that the ALPS index correlates with the clinical severity of Alzheimer’s disease, and it remains robust across different MRI scanners [25]. Furthermore, the ALPS index has been associated with glymphatic clearance based on glymphatic MRI after intrathecal gadolinium administration [26]. As a result, the DTI-ALPS method has emerged as a valuable tool for assessing glymphatic function, and its clinical validity has recently been further demonstrated in neurological disorders such as Parkinson’s disease, sleep disorders, traumatic brain injury, and stroke [27–30]. However, the changes in glymphatic function in cerebral palsy due to periventricular leukomalacia remain poorly understood, and the ALPS methodology has not been applied to assess glymphatic system function in this context.

In this study, we aim to measure the DTI-ALPS index in preterm children with bilateral spastic CP due to PVL. Additionally, we will construct a mediation model to explore the relationships among the DTI-ALPS index, brain lesion burden, and hand dysfunction in children with CP. By investigating these associations, our study aims to provide further insight into the potential role of impaired intracerebral glymphatic function in the pathogenesis of hand dysfunction in patients with PVL.

## Materials and methods

### Study participants

**Fig. 1:**
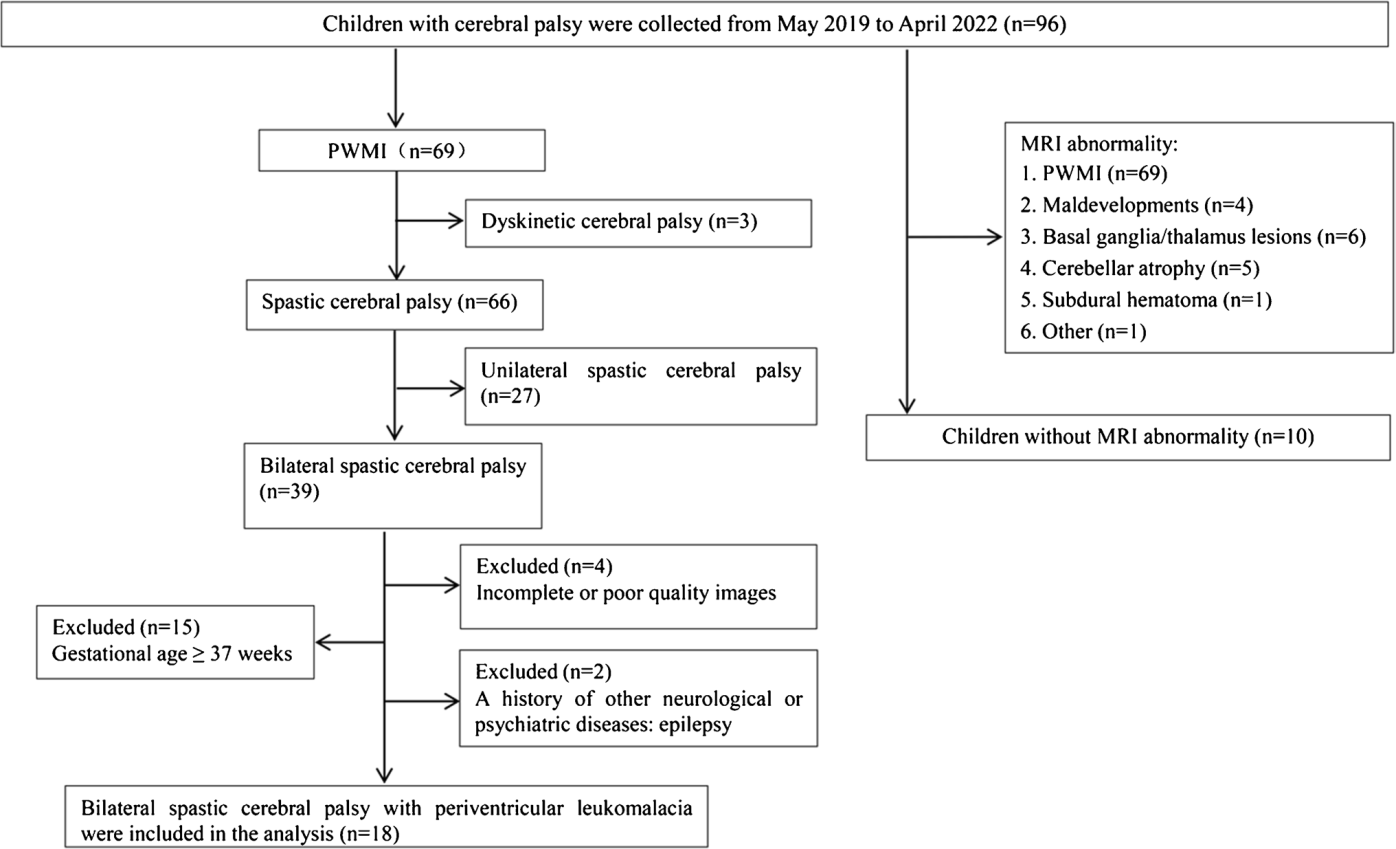
Flowchart of the inclusion process for children with bilateral spastic cerebral palsy. PWMI, periventricular white matter injury

This study was approved by the Ethical Committee and was in accordance with the World Medical Association (Declaration of Helsinki). Informed consent was obtained from the parents or guardians of all enrolled children. A total of 96 consecutive children diagnosed with cerebral palsy at our hospital from May 2019 to April 2022 were enrolled in this study. The inclusion criteria were a clinical diagnosis of spastic CP [31] and conventional MRI showing signs of periventricular white matter injury, which is characterized by bilateral periventricular T2-weighted hyperintensity, lateral ventricle dilatation, and thinning of the corpus callosum. The exclusion criteria were gestational age ≥ 37 weeks, a history of other neurological or psychiatric diseases, incomplete clinical or MRI data, and poor MRI image quality. Finally, 18 children with bilateral spastic CP due to PVL were included. A flowchart of patient inclusion is shown in Fig. 1.

Twenty-nine age- and sex-matched children with typical development (TD) were recruited at the same time. The inclusion criteria for the TD group were full term (37–42 gestational weeks), no abnormalities on routine MRI, no history of perinatal ischemia or hypoxia encephalopathy, no neurological or psychiatric diseases, and no visual or hearing impairments.

Hand function of the children with CP was assessed using the Manual Ability Classification System (MACS) within 3 days of the MR examination. The MACS classification system evaluates CP children’s ability to manipulate objects during daily life [32]. The MACS is a reliable and stable ordinal scale that consists of five levels, with a higher grade indicating worse clinical performance ability.

### Imaging acquisition and evaluation

MRI data were collected using the GE Signa HDx 3.0T MR scanner. The participants were instructed to remain still in the scanner with noise-canceling earplugs, and sponges were placed on both sides of the head to reduce motion artefacts. For uncooperative children, oral 10% chloral hydrate was administered (0.25–0.5 ml/kg; the maximum dose of 10 ml/d), and guardians were informed about potential adverse reactions and precautions. The MRI protocols consisted of three-dimensional T1-weighted imaging (3D-T1), T2-weighted imaging (T2WI), T2 fluid-attenuated inversion recovery (T2-FLAIR), DTI, and susceptibility-weighted imaging (SWI) sequences. The imaging parameters were as follows: (1) 3D-T1: repetition time = 7.8 ms, echo time = 3.0 ms, inversion time = 450 ms, field of view = 256 mm × 256 mm, slice thickness = 1 mm, slice spacing = 0 mm, flip angle = 15°; (2) T2WI: repetition time = 4480 ms, echo time = 120 ms, field of view = 240 mm × 240 mm, slice thickness = 5 mm, slice spacing = 6.5; (3) T2-FLAIR: repetition time = 7500 ms, echo time = 140 ms, field of view = 240 mm × 240 mm, slice thickness = 3.0 mm, slice interval = 1.5 mm; (4) DTI: repetition time = 12,500 ms, echo time = 84 ms, field of view = 240 mm × 240 mm, slice thickness = 2.5 mm, in-plane resolution = 2.2 mm × 2.2 mm, matrix = 96 × 96, *b* value = 0 or 1000, diffusion direction = 64; (5) SWI: repetition time = 39 ms, echo time = 5.5: 6: 35.5 ms, field of view = 230 mm × 230 mm, slice thickness = 2 mm, matrix = 384 × 382, flip angle = 17°, with in-plane spatial resolution = 0.6 mm × 0.6 mm.

Based on our previous research and literature reports, we examined anatomical regions significantly associated with motor function in children with CP [6, 8, 33, 34]. These regions include the periventricular white matter area, cerebral peduncle, posterior limb of the internal capsule, centrum semiovale, corpus callosum, precentral gyrus, cingulate gyrus, thalamus, and putamen. The axial T2-FLAIR sequence was primarily used to score the imaging manifestations in CP children, where the main white matter areas around the lateral ventricle appear as hyperintensity, cystic degeneration, and volume reduction. Each region was given a score of 1 for unilateral lesions, 2 for bilateral lesions, and 0 for normal (except for the corpus callosum, which was scored 1 for lesions and 0 for normal). Finally, we developed a preliminary scoring system consisting of 11 scoring items for the imaging manifestations in CP (Table 1). The Evans index was also measured from a single T1-weighted axial image reconstructed from MRI images. Two radiologists with more than 5 years of MRI experience independently rated the images. Any disagreement between the two raters was resolved by consensus.

**Table 1:** Brain lesion burden scoring system

Scoring items		Scoring criteria
Periventricular white matter	High signal	Normal: 0 point Unilateral lesion: 1 point Bilateral lesions: 2 points
	Cystic degeneration	
	Volume reduction	
Major white matter fiber damage	Centrum semiovale	
	Posterior limb of internal capsule	
	Cerebral peduncle	
	Corpus callosum	
Major cortical damage	Precentral gyrus	
	Cingulate gyrus	
Deep gray matter damage	Thalamus	
	Putamen	

### Quantification of DTI-ALPS

Data were analyzed using the FMRIb Software Library (FSL; www.fmrib.ox.ac.uk/fsl) for DTI preprocessing. The processing flow included correction for eddy currents, skull stripping, and fitting of diffusion tensors to generate fractional anisotropy (FA) and mean diffusion coefficient maps [35].

**Fig. 2:**
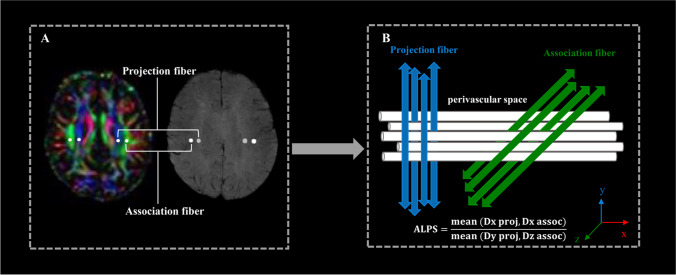
Schematic diagram of the processing of the DTI-ALPS method. **A** Color-coded fractional anisotropy (FA) map at the lateral ventricle body level demonstrating projection fibers (*y*-axis, blue) and association fibers (*z*-axis, green). ROIs are drawn on left projection fibers and association fibers following the course of the medullary vessels using the reference information obtained from SWI. **B** The ALPS index was calculated after extracting the diffusivity of each ROI along the *x*-, *y*-, and *z*-axes, respectively

The method for calculating DTI-ALPS is summarized in Fig. 2. The DTI-ALPS index was calculated as the ratio of the mean diffusivity along the *x*-axis of the projection fiber and association fiber regions of interest (ROIs) to the mean diffusivity along the *y*-axis and *z*-axis of the same ROIs (3 mm^3^) [24]. The ROIs were drawn on the color-coded FA map at the same level as the reference SWI in the left hemisphere since all the participants included in the analysis were right-handed according to previous research [36], while avoiding significantly damaged tissue:

$$\textrm{ALPS}=\frac{\textrm{mean}\ \left(\textrm{Dx}\ \textrm{proj},\kern0.5em \textrm{Dx}\ \textrm{assoc}\right)}{\textrm{mean}\ \left(\textrm{Dy}\ \textrm{proj},\textrm{Dz}\ \textrm{assoc}\right)}$$

### Statistical analysis

All statistical analyses were performed using SPSS (Version 20.0; IBM, Armonk, New York, USA) software. First, the Kolmogorov–Smirnov or Shapiro–Wilk test and Levene’s test were used to assess the normality and homoscedasticity of continuous data separately. Two independent sample *t*-tests were used to compare the age, gestational age, and DTI-ALPS index between the CP group and the control group. The chi-square test was performed on the gender of the two groups.

To investigate the relationship between brain lesion burden, hand dysfunction, and glymphatic function in children with CP, a mediation analysis was conducted. The mediation analysis aimed to determine whether glymphatic injury could mediate the association between brain lesion burden and hand dysfunction. In this analysis, brain lesion burden was considered an independent variable (*X*), MACS indicator was a dependent variable (*Y*), and the DTI-ALPS index was a mediator variable (*M*), while controlling for Evans index, gender, and age. The significance of the mediating effect was assessed by the PROCESS 4.1 software package based on SPSS 20.0. This method generates a bootstrap confidence interval, and the mediating effect is considered significant when the interval does not contain 0. The mediating effect size was obtained by calculating the proportion of the indirect effect to the total effect (a*b/*c*×100%).

## Results

### Demographic and clinical information

The study included 18 children with bilateral spastic CP due to PVL (10 female; 8.37 ± 2.47 years old) and 29 TD (15 female; 8.53 ± 2.71 years old). There was no significant difference in age (*P* = 0.80) or sex ratio (*P* = 0.68) between the children with the CP group and the TD group. The gestational age of the children with CP was significantly lower than that of the children in the TD group (*P* < 0.001). For more detailed clinical information about the participants, please refer to Table 2.

**Table 2:** Demographic and clinical characteristics

Characteristics	CP (*n*=18)	TD (*n*=29)	*P* value
Gender (male/female)	8/10	14/15	0.68
Handedness (L/R)	0/18	0/29	
Age at MRI (year)	8.37 ± 2.47	8.53 ± 2.71	0.80
Gestational age (week)	32.21 ± 2.58	39.50 ± 1.08	<0.001
Evans index	0.25 ± 0.02	0.24 ± 0.02	0.003
MACS (I/II/III/IV/V)	8/3/3/3/1		

*CP* cerebral palsy, *TD* typical development, *L* left, *R* right, *MACS* Manual Ability Classification System

### Brain lesion burden scores

**Fig. 3:**
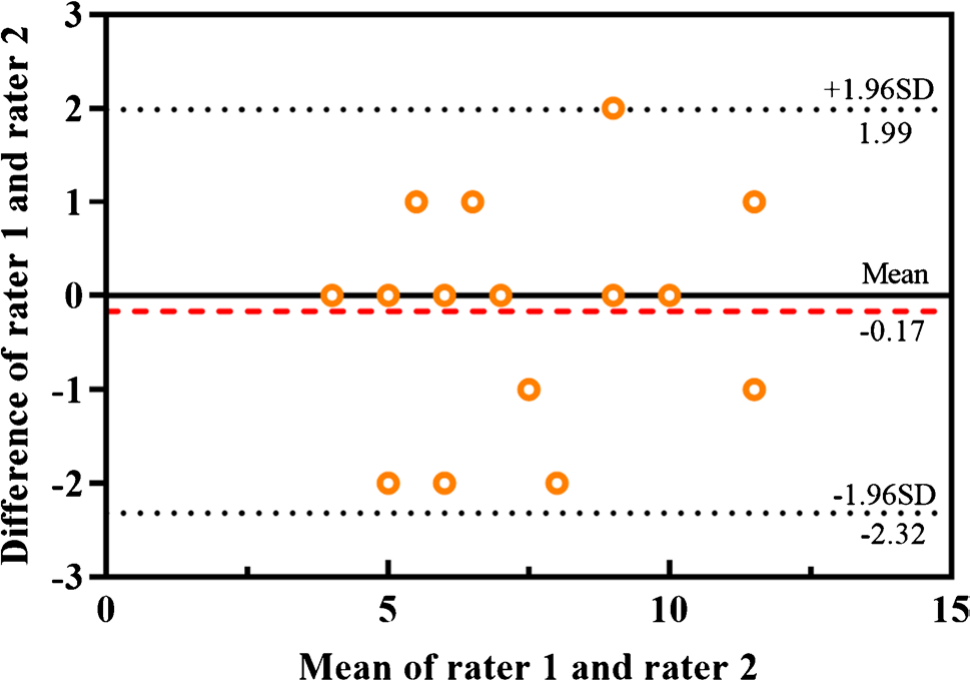
Bland-Altman plot. Consistency of rater 1 and rater 2 for the determination of the brain lesion burden score. The red dotted line represents bias; the black dotted lines represent the upper and lower limits of agreement (±1.96 SD)

The brain lesion burden scores in CP were distributed as follows: score of 4, 4 (22.2%); score of 5, 2 (11.1%); score of 6, 3 (16.7%); score of 7, 4 (22.2%); score of 9, 1 (5.6%); score of 10, 2 (11.1%); score of 11, 1 (5.56%); score of 12, 1 (5.56%). The interrater agreement for brain lesion burden was excellent (ICC_inter_: 0.91, *P* < 0.001). The Bland-Altman analysis for interrater agreement is shown in Fig. 3.

### Group differences in the ALPS index

**Fig. 4:**
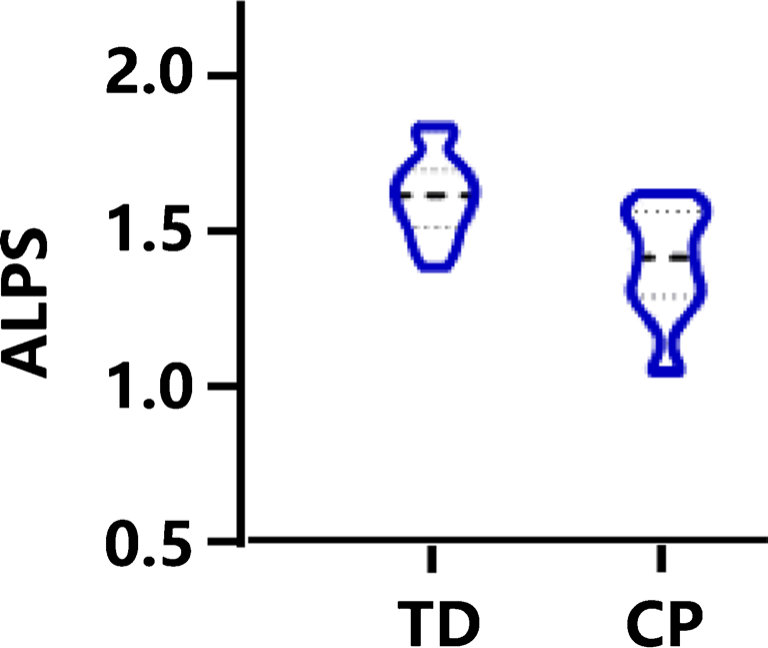
Violin plot showing differences in ALPS index between children with CP and TD. TD, typical development; CP, cerebral palsy

The ALPS index of children with CP was significantly lower than that of the controls (1.448 ± 0.249 vs. 1.625 ± 0.142, *t* = 3.131, *P* = 0.003) (Fig. 4).

### Mediation analysis

**Fig. 5:**
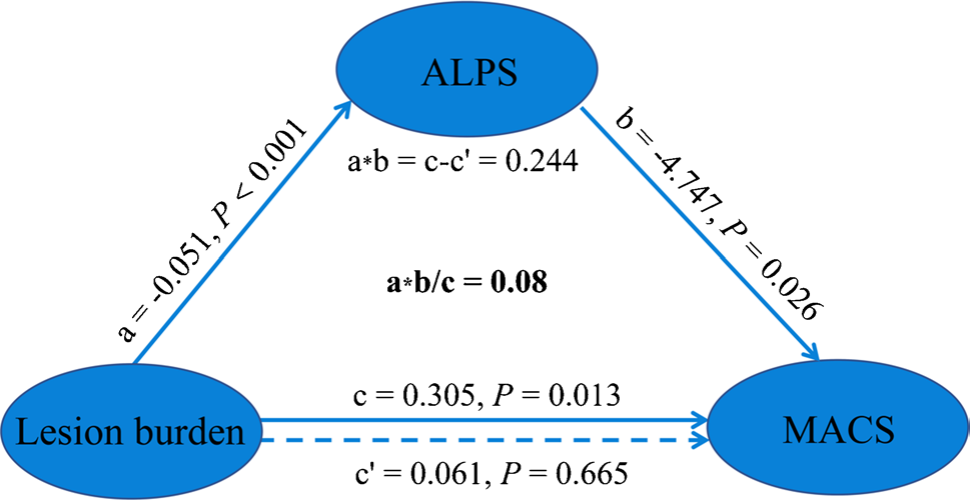
Mediation analysis. In the mediation analysis, the independent factor was lesion burden and the dependent variable was the MACS indicator, while ALPS served as the proposed mediator. ALPS, along the perivascular space; MACS, Manual Ability Classification System

The mediation analysis showed that the ALPS index completely mediated the relationship between brain lesion burden and MACS level in children with CP (90% CI: 0.004, 0.410; c′ = 0.061, *P =* 0.665; Fig. 5). The mediating effect size was 80%.

## Discussion

The present study employed a novel approach to investigate the function of the glymphatic system in children with CP using a noninvasive index analysis of DTI-ALPS. The main findings are as follows: (A) children with CP exhibited a significantly lower ALPS index compared to children with TD; (B) the lower ALPS index fully mediates the effect of brain lesion burden on hand dysfunction in children with CP.

Although T2 hyperintensities are commonly used as markers of visible brain lesions in cerebral palsy (CP) and are associated with structural abnormalities, it is important to note that not all brain lesions or impairments are visible on T2-weighted images. The DTI-ALPS index, on the other hand, provides a quantitative measure of glymphatic function, which is related to the clearance of interstitial fluid and waste products from the brain [37]. The glymphatic system is a recently discovered waste clearance system in the brain that relies on the movement of cerebrospinal fluid along perivascular spaces to remove metabolic waste products [15]. Impairment in glymphatic function can lead to the accumulation of waste products and potentially contribute to neurological disorders [27–30]. While T2 hyperintensities provide valuable information about visible brain lesions, the DTI-ALPS index offers insights into the underlying glymphatic dysfunction, which may not be limited to regions with visible abnormalities. By examining the glymphatic function beyond T2 hyperintensities, we aimed to provide a more comprehensive understanding of the pathophysiological mechanisms in CP.

In this study, the children with CP due to PVL demonstrated significantly lower ALPS index values outside regions with T2 hyperintensities, indicating impaired glymphatic function and reduced interstitial fluid clearance. Preterm infants are developmentally immature and susceptible to oxidative stress and proinflammatory mediators, which can induce blood-brain barrier disturbance by inducing endothelial tight junctions and basement membranes [38, 39]. The perivascular space is not only an important glymphatic pathway, but also an intrinsic component of the central nervous system parenchymal vasculature; any process that disrupts blood-brain barrier integrity may impair regional glymphatic function [40]. Moreover, the blood-brain barrier disruption could also lead to abnormal cerebrospinal fluid circulation. The glymphatic system is an essential regulator of immune cell trafficking, immunosurveillance, and clearance of macromolecules [41]. Prior studies have found that periventricular leukomalacia in preterm infants is often accompanied by increased cerebrospinal fluid volume [42, 43]. Immune cells and serum substrates in the cerebrospinal fluid might infiltrate and accumulate in the perivascular space through the compromised blood-brain barrier, thus disrupting the balance between increased metabolite secretion and decreased clearance.

Glymphatic clearance is dependent on AQP-4 channels that are densely expressed on astrocyte end-feet adjacent to cerebral capillaries. It has been shown that glial cell proliferation in response to inflammatory signals can disrupt the glymphatic system by altering AQP-4 expression and polarity in animals [21, 44]. Reactive astrocyte gliosis is a major pathologic alteration in periventricular leukomalacia. AQP-4 may function abnormally after white matter injury in preterm infants and contribute to the brain clearance reduction. In addition, arterial pulsation as a driver of perivascular blood flow contributes to the glymphatic function [45]. Since the blood vessels responsible for cerebral white matter blood supply are incompletely developed, arterial pulsation compromise tends to appear in preterm infants with periventricular white matter injury [5], which is somehow related to the reduced glymphatic system efficiency. The above-mentioned mechanisms might contribute to the glymphatic system damage in PVL, which is also consistent with our results. Thus, quantifying ALPS parameters may help to further explore the relationship between glymphatic function and disease.

The significantly reduced glymphatic function revealed by DTI-ALPS in this study played a mediating role between brain lesion burden and hand dysfunction in children with CP, highlighting its importance in hand motor function in CP. Previous studies have mainly focused on quantifying brain lesion burden and its association with motor function outcomes in CP, with ample evidence supporting a significant negative correlation between them [14, 46]. However, this relationship remains controversial as not all children with PVL exhibit neurological abnormalities, and routine magnetic resonance examinations may not detect structural abnormalities in some patients with spastic diplegia [47]. These conflicting results suggest the presence of other explanatory variables, which can be well explained by the mediating role of glymphatic function identified in this study.

From a developmental perspective, key neuronal damage in the thalamus, cerebral cortex, and basal ganglia in preterm children is known to be commonly associated with PVL after prenatal hypoxic-ischemic injury [48, 49]. The corticospinal, which run through the periventricular white matter, connects the motor-related cortex with the spinal cord. This region is often the most affected during pathological injury, leading to the development of spastic CP. In addition, axonal dysfunction caused by subplate neuronal damage could trigger decreased white matter volume in transsynaptic sensorimotor cortex and thalamic/basal ganglia [50]. The thalamocortical projections are essential in determining hand motor function [51]. Therefore, the factors inducing PVL in preterm children may increase the relative accumulation of metabolic waste by blocking the glymphatic circulation, resulting in the disruption of sensorimotor pathways and hand dysfunction.

Despite the novel findings in this study, limitations are worth noting. Firstly, the sample size is relatively small. Future research with larger samples is needed to validate these findings. Secondly, this study uses an indirect measure of glymphatic function, brain clearance in CP also probably depends on other well-established clearance mechanisms (via BBB, local degradation, etc.), so animal models may be needed for validation in the future. Thirdly, the cross-sectional study design prevented us from elucidating the underlying causation between glymphatic system damage and brain injury burden; longitudinal studies are expected to provide further insights into the role of glymphatic function in the development of PVL into CP.

## Conclusion

In conclusion, this study employed a diffusion-based noninvasive imaging method to assess glymphatic system function, providing new insights into the neural basis of hand dysfunction in CP due to PVL and highlighting the important role of glymphatic impairment in these patients. The results suggest that PVL may affect hand function in children with CP by disrupting drainage associated with glymphatic function. Further research is needed to confirm these findings and explore potential interventions targeting the glymphatic system to improve motor outcomes in children with CP.

## Data Availability

Data generated or analyzed during the study are available from the corresponding author by request.
